# Comprehensive Phytochemical and Biological Evaluation of *Boerhavia elegans* Extracts: Anticancer and Antioxidant Activities in Various Solvent Systems

**DOI:** 10.1155/ianc/3806655

**Published:** 2026-04-02

**Authors:** Tahreer M. Al-Raddadi, Saleh O. Bahaffi, Lateefa A. Alkhateeb, Abdulaziz A. Kalantan, Ahmed M. Adam, Torki Zughaibi, Ehab M. M. Ali

**Affiliations:** ^1^ Chemistry Department, Faculty of Science, King Abdulaziz University, Jeddah, 21589, Saudi Arabia, kau.edu.sa; ^2^ Chemistry Department, Al-Qunfudah University College, Umm Al-Qura University, Makkah, Saudi Arabia, uqu.edu.sa; ^3^ King Fahd Medical Research Center, King Abdulaziz University, Jeddah, 21589, Saudi Arabia, kau.edu.sa; ^4^ Biochemistry Department, Faculty of Science, King Abdulaziz University, Jeddah, 21589, Saudi Arabia, kau.edu.sa; ^5^ Department of Medical Laboratory Sciences, Faculty of Applied Medical Sciences, King Abdulaziz University, Jeddah, 21589, Saudi Arabia, kau.edu.sa; ^6^ Division of Biochemistry, Chemistry Department, Faculty of Science, Tanta University, Tanta, 31527, Egypt, tanta.edu.eg

**Keywords:** antioxidant activity, *Boerhavia elegans*, cytotoxicity, molecular docking, total flavonoids content, total phenolic content, UHPLC

## Abstract

*Boerhavia elegans* has long‐held medicinal importance, yet its comprehensive phytochemical and biological characterization remains limited. This study provides the first integrated analysis combining HPLC, GC–MS, and molecular docking to correlate the chemical composition of *B. elegans* with its antioxidant and cytotoxic activities across different plant parts and solvent systems. Antioxidant assays revealed that polar leaf extracts exhibited the highest activity, with a DPPH IC_50_ value of 16.73 μg/mL. The methanolic stem extract showed the greatest phenolic content (25.89 mg GAE/100 mg), and the methanolic seed extract had the highest flavonoid yield (6.717 mg QE/100 mg). Ethyl acetate favored flavonoid extraction from the stem (18.29 mg QE/100 mg), while diethyl ether was most effective for the roots (10.21 mg QE/100 mg). UHPLC–DAD identified 15 phenolic compounds with detection limits ranging from 0.47 to 2.20 μg/mL. Cytotoxicity assays demonstrated strong inhibitory effects, with the methanol leaf extract showing 82.5% inhibition of HepG2 cells and the ethyl acetate root extract inhibiting 83.55% of MCF‐7 cells. The hexane leaf extract produced the highest inhibition (88.8%) of MDA‐MB‐231 cells. GC–MS analysis revealed bioactive molecules such as phytol, stigmasterol, and hexadecanoic acid, which were further validated by molecular docking interactions with vimentin and BCL‐2 proteins, suggesting potential anticancer mechanisms.

## 1. Introduction

Throughout history, plants have been a vital source of medicinal remedies, with traditional uses highlighting their potential for discovering new therapeutic drugs [1, 2]. The effectiveness of plant treatments relies on the specific phytochemical compounds they contain, which can exert various physiological effects. Consequently, phytochemical screening methods have been employed to identify these bioactive compounds, which may serve as the basis for the development of new pharmaceuticals [3, 4].

Free radicals are molecules with one or more unpaired electrons within their atomic or molecular structures [5]. Free radicals react with biological molecules to cause cell destruction, which can lead to diseases [6, 7].

Antioxidants are essential compounds that can protect organisms from damage caused by oxidative stress induced by free radicals [8]. Unlike synthetic antioxidants, naturally derived antioxidants have recently gained more attention because of their ability to combat free radicals in the human body without causing harmful side effects [9]. Recently, there has been growing interest in the commercialization of naturally occurring antioxidants [10].

Phenolic compounds are secondary metabolites produced in plants from shikimic acid and pentose phosphate through phenylpropanoid metabolism [11]. They contain benzene rings with one or more hydroxyl substituents and range from simple phenolic molecules to highly polymerized compounds [12–14]. Recent research has identified numerous antioxidants and radical scavenging compounds in plants, fruits, vegetables, and medicinal herbs. These include phenolic compounds, flavonoids, vitamins, and terpenoids, all of which exhibit strong antioxidant potential [15–17].

The medicinal efficacy of plants in the *Boerhavia* genus has long been recognized in various traditional medicine systems worldwide. *Boerhavia* species have therapeutic benefits in Indian disciplines such as Ayurveda, Siddha, and Unani. Botanists categorize *elegans* under the genus Boerhavia, which encompasses a diverse range of over 100 species, and assign it to the Nyctaginaceae family. This blooming plant is most commonly found in tropical and subtropical zones, although it also grows in Western India, Iran, Pakistan, Oman, and Saudi Arabia [18, 19]. The plant genus *Boerhavia* has increasingly interested physiochemists as past studies have demonstrated its wide range of pharmacological and biological effects, signifying potential therapeutic applications. Research has found that a methanolic extract of *Boerhavia diffusa* leaves exhibits antibacterial properties and noteworthy inhibition against *Staphylococcus aureu*s, a known pathogenic bacterium [20]. According to traditional medicine systems, *Boerhavia elegans* has been used to treat various health conditions, including painful menstruation, urinary tract infections, intestinal infections, inflammation, jaundice, and overall weakness or fatigue [21]. Cancer is a leading global health challenge, second only to cardiovascular diseases in terms of mortality rates, and poses significant risks worldwide. Standard treatments such as chemotherapy and radiotherapy often damage normal cells, carry high financial costs, and can lose effectiveness over time. Complementary alternative treatments, particularly those derived from medicinal plants, have shown promise in treating various cancers, including lung cancer, by inducing cancer cell death, inhibiting tumor growth, and targeting specific cellular pathways. The interest in plant‐derived compounds as cancer treatments is increasing, with research focusing on phytochemicals that can effectively target tumors while minimizing harm to healthy cells [22–24].

In addition to evaluating therapeutic efficacy, it is equally important to assess the toxicological profile and environmental safety of plant‐derived bioactive compounds to ensure their safe application in pharmaceutical and biomedical fields. Recent toxicological investigations have emphasized the need for comprehensive safety assessments of natural products prior to their clinical or industrial utilization [25]. The traditional drug development process is known for its complexity, high cost, and time‐consuming nature. However, the advent of computational approaches, such as molecular docking, has revolutionized this field. This technology, which allows for the rapid screening of drug candidates from libraries, has become a key player in drug research [26–29]. Computational methods have revolutionized pharmaceutical discovery by providing essential tools for drug development. These techniques significantly reduce costs and enhance the efficiency of discovering and creating new medications [30, 31]. Literature indicates that the biological activity of *B. elegans* remains largely unexplored. Therefore, this study was designed to comprehensively evaluate the therapeutic potential of *Boerhavia elegans* by assessing its antioxidant, phenolic, flavonoid, antimicrobial, and cytotoxic activities using extracts obtained with solvents of varying polarity. The choice of solvent is a crucial factor influencing the yield and composition of secondary metabolites that determine biological activity. Understanding these solvent‐dependent variations is essential for optimizing extraction efficiency and maximizing the recovery of bioactive constituents. Other parts of the plant, particularly the stem, have already been investigated in previous studies [32], whereas the present work focuses on the seeds, which are traditionally consumed as a food component in local cuisines but remain underexplored in terms of their phytochemical composition and biological effects. Moreover, the polyphenolic profile of *B. elegans* was analyzed using high‐performance liquid chromatography with diode‐array detection (HPLC–DAD) to identify and quantify the major phenolic compounds responsible for its observed bioactivities.

## 2. Materials and Methods

### 2.1. Reagents and Chemicals

HPLC‐grade solvents such as methanol, trifluoroacetic acid (TFA), acetonitrile, diethyl ether, and ethyl acetate were obtained from Fisher Scientific UK Ltd., and Folin–Ciocalteu’s phenol reagent was purchased from Sigma‐Aldrich. Sodium carbonate, potassium hexacyanoferrate, and ferric chloride were obtained from BDH Chemicals Ltd. (England), and 1,1‐diphenyl‐2‐picrylhydrazyl (DPPH) radical was supplied by Sigma‐Aldrich (St. Louis, MO, USA). Standard (purity > 99.0%) phenolic compounds for HPLC analysis are as follows: gallic acid, coumalic acid, chlorogenic acid, vitamin C, vanillic acid, caffeic acid, syringic acid, ellagic acid, *p*‐coumaric acid, rutin, ferulic acid, myricetin, rosmarinic acid, resveratrol, quercetin, and *trans*‐cinnamic acid. The Dulbecco’s modified Eagle medium (DMEM) high‐glucose media, fetal bovine serum (FBS), penicillin/streptomycin antibiotic, and 0.25% EDTA trypsin were supplied by Life Technologies Gibco. MTT (3‐(4,5‐dimethylthiazol‐2‐yl)‐2,5‐diphenyltetrazolium bromide) and dimethyl sulfoxide (DMSO) were supplied by Sigma‐Aldrich.

### 2.2. Preparation of Extracts

#### 2.2.1. Material and Extracts

*Boerhavia elegans* is imported from the Hadramout region of South Yemen. For preparation, the seeds, stems, leaves, and roots were freeze‐dried (Il‐shin Bio Base) before use during extraction. Thirty grams of *B. elegans* seeds, stems, leaves, and root powder were extracted using 300 mL of different solvents through a 24‐h soaking process. Various solvents were extracted, including hexane, ethyl acetate, diethyl ether, and methanol. After filtration to remove plant debris, the solvent was removed using a rotary evaporator system (Stuart RE300 Rotary Evaporator).

#### 2.2.2. Preparation of HPLC Samples

The *B. elegans* plant was dissected into four main parts: seeds, leaves, stems, and roots. Approximately 8 g of each dried and powdered sample was subjected to extraction using 50 mL of methanol in a flask within a sonication apparatus for 3 h at 50°C. This initial methanol extract was further processed using three methods to analyze phenolic substances using HPLC–PDA. First, a portion of the methanol extract was directly analyzed without additional treatment. Second, a selective extraction method is employed. The remaining methanol extract was evaporated to dryness using a rotary evaporator at 50°C. The resulting residue was then re‐dissolved in distilled water and subjected to liquid–liquid extractions using diethyl ether and ethyl acetate, performed three times consecutively. The organic phases were combined, evaporated under reduced pressure at 40°C using a rotary evaporator, weighed, and finally dissolved in methanol for HPLC analysis. The final method involved acidic hydrolysis. The remaining methanol extract was evaporated to dryness using a rotary evaporator. This residue was then dissolved in a 2 M hydrochloric acid solution and hydrolyzed for 2 h at a constant temperature of 90°C. After cooling, the acidic mixture underwent liquid–liquid extractions, as described in the second method. The organic solvents were evaporated, and the residue was weighed and dissolved in methanol for subsequent HPLC analysis [33].

### 2.3. Apparatus and Chromatographic Conditions

Extracts were analyzed using a Shimadzu UFLC equipped with a 20A control processing unit, LC‐20AD solvent delivery pump, 20As degassing unit, and photodiode array detector. The system was operated in low‐pressure gradient mode (LC‐20DA) at a flow rate of 1.0 mL/min, and detection was performed at 245, 278, and 300 nm (Figure 1). The column used was a Waters X Bridge BEH C18 Column, with 5‐μm packing, an internal diameter of 4.6 mm, and a length of 250 mm. The column oven temperature was set to 25°C. The injection volume was 10 μL. The elution solvents were 0.1% TFA in water (A) and acetonitrile (B). The analytes were eluted according to the following gradient: 0–8 min linear gradient from 10% to 25% B; 8–16 min 25% B; 16–25 min 45% of B; 25–28 min 45% B; and 28–45 min 80% B. Following a duration of 45 min, the column equilibration process comprised two sequential steps: from 45 to 46 min, 100%–10% B, and 45–60 min, 10% B [34]. PDA absorption spectra of the standards and samples were recorded within a wavelength range of 190–400 nm. All solvents were filtered using 0.5‐mm filters from Millipore and degassed via ultrasonication.

FIGURE 1HPLC chromatograms of a standard mixture of analytes at (a) 254 nm, (b) 287 nm, and (c) 300 nm.

**Figure:**
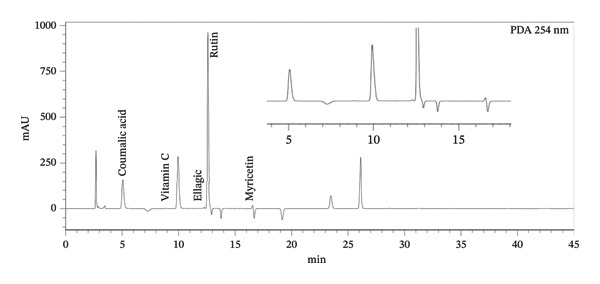
(a)

**Figure:**
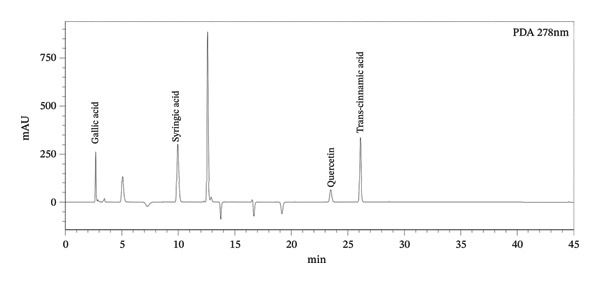
(b)

**Figure:**
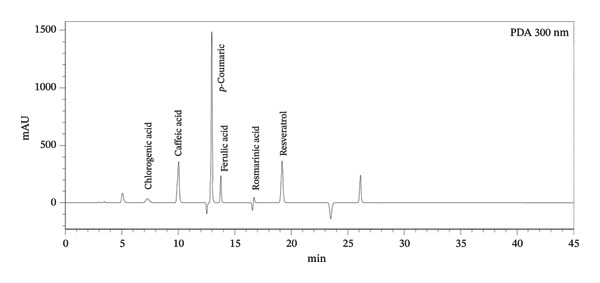
(c)

### 2.4. Scavenging of Free Radical (DPPH) Assay

Using DPPH, the plant extracts were tested for their capacity as free radical scavengers, with some modifications [35]. The DPPH assay was conducted using *B. elegans* extracts at varying concentrations (10, 8.75, 7.5, 6.25, 5, 3.75, 2.5, and 1.25 μg/mL) and placed in test tubes (50 μg). Then, 0.03 g was dissolved in 100 mL of methanol to prepare a stock solution. Next, 2 mL of DPPH was added to each test tube. After incubation at room temperature for 10 min, the absorbance of the resultant solution was measured at 517 nm. The tube containing only 3.0 mL of DPPH without extract was identified as Ao, whereas the reference blank was methanol. Ascorbic acid (1.25–10 μg/mL) generated a standard calibration curve (*y* = 9.9232*x* + 306,987 − *r* = 0.91). DPPH scavenging activity percentage was determined using the following equation:

(1) DPPH scavenging %=Ao−AsAo×100,

where Ao is the absorbance of the blank, As is the absorbance of the sample or standard at 517 nm, and IC_50_ is the amount (mg) of sample in 1 mL of solution needed to reduce the DPPH to 50%.

In addition, the Antioxidant Activity Index (AAI) was calculated and classified according to [36] standardize antioxidant comparisons among extracts, using the following equation:

(2) AAI=DPPHworkingIC50.

### 2.5. Phytochemical Investigations

#### 2.5.1. Determination of Total Phenolic Content (TPC)

The TPC was evaluated using a Folin–Ciocalteu colorimetric assay with minor modifications [37]. In this essay, 1 mL of Folin–Ciocalteu reagent was added to the sample, followed by 1 mL of 7.5% sodium carbonate after a certain period. An absorbance was measured at 760 nm. Gallic acid (0–100 μg/mL) generated a standard calibration curve (*y* = 0.0113*x* − 0.0343, *r* = 0.990). An equation was established from the standard gallic acid calibration graph to assess the TPC in milligrams of gallic acid equivalent (mg GAE)/100 mg of extract.

#### 2.5.2. Determination of Flavonoid Content

Total flavonoid content (TFC) was estimated using the AlCl_3_ reagent [38]. Next, 200 μL of each *B. elegans* extract and 800 μL of methanol were mixed with 1000 μL of AlCl_3_ 2% in methanol. After 10 min of incubation, the absorbance was read at 415 nm against a blank of 200 μL plant extract and 1800 μL of methanol without AlCl_3_. Quercetin (0–50 μg/L) was used to plot the standard calibration curve (*y* = 0.0246 *x* − 0.0079, *r* ^2^ = 0.999). The results were presented as μg of quercetin equivalent (mg QE)/100 mg of extract.

#### 2.5.3. Preparation of Mixed Standard Solutions

A mixed standard solution with a concentration of 1 mg/mL was prepared by precisely weighing 25.00 mg of each phenolic compound in a 25‐mL volumetric flask, then diluting to volume with absolute methanol. Calibration curves for the standards were generated in the 5–50 μg/mL concentration range by diluting an appropriate volume of the stock solution with methanol in a volumetric flask. All solutions were stored at 4°C before injection. The identification of compounds was based on retention time (RT), UV absorption spectrum, and the addition of standards to the analyzed extract samples. Fifteen phenolic compounds (gallic acid, coumalic acid, chlorogenic acid, vitamin C, caffeic acid, syringic acid, ellagic acid, rutin, *p*‐coumaric acid, ferulic acid, myricetin, rosmarinic acid, resveratrol, quercetin, and *trans*‐cinnamic acid) were used to identify the phenolic compounds in methanol extracts of the four parts of the plant. Each extract sample was analyzed in triplicate (*n* = 3) under identical chromatographic conditions to ensure reproducibility. The data reported in the tables represent the mean ± standard deviation (SD) of these independent measurements, and the relative standard deviation (RSD) was maintained below 2%, confirming the precision and reliability of the HPLC results. The limit of detection LOD and limit of quantification (LOQ) were determined to identify and quantify phenolic compounds in the extracts. According to [39], the LOD and LOQ were calculated [39].

(3) LOD=3Syx/b,

(4) LOQ=10Syx/b,

where Sy/*x* is the SD of the y‐residual and *b* is the slope of the calibration plot.

### 2.6. Gas Chromatography–Mass Spectrometry (GC/MS) Analysis of *B. elegans* Seed Extract and Molecular Docking Study

#### 2.6.1. Extraction and Preparation of Sample

The extraction method has been described by Alraddadi et al. [40]. The *B. elegans* seed sample was prepared using an open column with 20% methanol and ethyl acetate, yielding 0.35 mg of oily material. The *B. elegans* extract in methanol was analyzed using the GC/MS technique to identify phenolic bioactive compounds.

#### 2.6.2. GC–MS

GC–MS analysis was conducted using a TSQ 8000 Evo MS instrument. Helium with a purity of 99.9995% was utilized as the carrier gas at a 1 mL/min flow rate. The transfer line temperature was set to 300°C, while the ion source temperature was maintained at 290°C. The run was scheduled to stop after 45 min. The filament/multiplier/dynode and chromatography filter were activated, with the peak width time set to 1 s. The tuned file emission current was utilized, and the last tuned detector gain was used with a multiplier of 1. The tune file electron energy was used with an electron energy of 70 eV. The NIST library mass spectra were used to identify and interpret the GC–MS mass spectra of the bioactive compounds.

#### 2.6.3. Molecular Docking of Some Bioactive Compounds in *B. elegans* Extract to Target Some Proteins in Cancer Cells

The chemical structures derived from extracts of *Boerhavia elegans* were downloaded and saved in the SDF format from the PubChem database (https://pubchem.ncbi.nlm.nih.gov/). The structures of vimentin and BCL2, which are overexpressed in various cancers, were obtained from the Protein Data Bank database (https://www.rcsb.org/). Both receptors were prepared before docking using BIOVIA Discovery Studio, in which water molecules and unwanted atoms were removed. PyRx 0.8 software was used to perform molecular docking between the ligands and protein macromolecules, and Discovery Studio Visualizer was employed to examine the results.

### 2.7. Evaluations of the IC50 of *B. elegans* Extracts in Human Cancer Cell Lines Using the MTT Assay

The cytotoxicity of *B. elegans* was tested on three human cancer cell lines: HepG2 (liver cancer), MCF7 (breast cancer), and MDA‐MB‐231 (triple‐negative breast cancer). These cell lines were obtained from the Tissue Culture Unit of the Department of Biochemistry at King Abdulaziz University. The cells were cultured in DMEM media supplemented with 10% FBS and 1% antibiotics and incubated in a CO_2_ incubator. To detach the cells, 0.25% EDTA trypsin was used, and cell counting was performed with 0.4% trypan blue. A total of 10^4^ cells per well were seeded into a 96‐well plate and incubated for 24 h. The cells were then treated with the crude hexane, diethyl ether, ethyl acetate, and methanolic extracts. Extracts were prepared by dissolving 20 mg in 1 mL DMSO, with concentrations ranging from 62 to 1000 μg/mL in media containing 5%–0.3% DMSO. After 48 h, the media were replaced with medium containing 0.5 mg/mL MTT and incubated in darkness at 37°C for 3 h. Finally, 100 μL of DMSO was added, and the absorbance was measured at 490 nm using an ELISA reader to calculate cell viability:

(5) % of viability=absorbance of treated cellabsorbance of untreated cells ×100.

### 2.8. Statistical Analysis

For statistical analysis, MS Excel software (Correl statistical function) was used to calculate gallic acid, quercetin, and ascorbic acid equivalents; determine the percentage of inhibition; and establish the linear regression equation. GraphPad Prism Software (Version 9.0, San Diego, CA, USA) was used to determine the *B. elegans* methanolic extract IC_50_.

## 3. Results

### 3.1. DPPH Assay

Figure 2 presents the IC_50_ values representing 50% DPPH radical scavenging activity of *Boerhavia elegans* extracts (roots, leaves, stems, and seeds) obtained using different solvents—methanol, ethyl acetate, diethyl ether, and hexane. These values indicate the extract or standard (ascorbic acid) concentration required to inhibit 50% of free radicals in each solvent system, where lower IC_50_ values correspond to stronger antioxidant activity.

FIGURE 2(a) A concentration of various extracts is needed to reduce the initial DPPH radical by 50%. (b) AAI of DPPH assay of analyzed *B. elegans* extracts.

**Figure:**
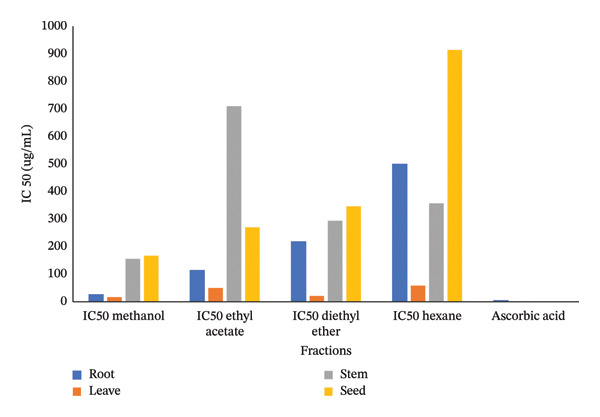
(a)

**Figure:**
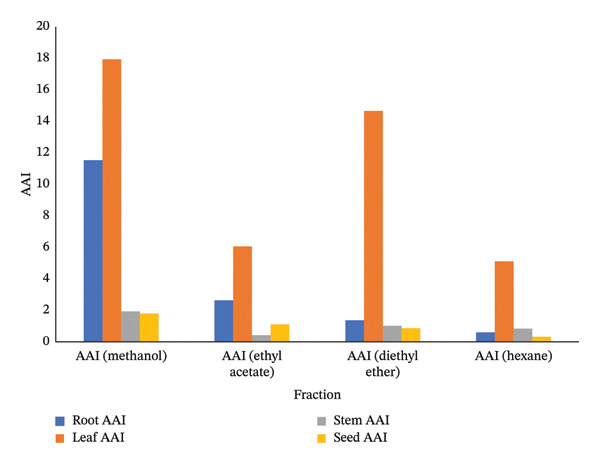
(b)

The methanolic root extract exhibited the highest antioxidant activity with an IC_50_ of 26.02 μg/mL, followed by the ethyl acetate (113.65 μg/mL), diethyl ether (218.3 μg/mL), and hexane (500 μg/mL) extracts. The leaf extract showed the most potent antioxidant capacity overall, with a methanolic IC_50_ of 16.73 μg/mL, and similarly strong activity with diethyl ether (20.46 μg/mL). In contrast, the stem and seed extracts showed higher IC_50_ values, indicating lower antioxidant potency. The stem extract exhibited IC_50_ values of 155.58 μg/mL (methanol), 710 μg/mL (ethyl acetate), 294.12 μg/mL (diethyl ether), and 357 μg/mL (hexane), whereas the seed extract showed the lowest activity, with a methanolic IC_50_ of 166.87 μg/mL.

Overall, methanol proved to be the most effective solvent for extracting antioxidants from *B. elegans*, particularly in the leaf extract, while hexane yielded the weakest results due to its low polarity.

The AAI results further confirmed that all *B. elegans* extracts possess measurable antioxidant potential, though the magnitude varied with both solvent type and plant part. The methanolic leaf extract exhibited the highest AAI value (17.9), followed by the diethyl ether leaf and methanolic root extracts, indicating very strong antioxidant activity. These findings correlate well with the high TPC and TFC in these extracts, highlighting the role of phenolics as key contributors to radical scavenging activity. Most other extracts demonstrated moderate to strong antioxidant potential, whereas the hexane and ethyl acetate stem extracts showed the weakest performance, reflecting their poor ability to extract polar antioxidant compounds. Overall, the AAI results corroborate the IC_50_ data and confirm that solvent polarity critically influences extraction efficiency, with methanol providing the highest recovery of bioactive constituents responsible for antioxidant activity in *B. elegans*.

### 3.2. TPC and TFC

Table 1 shows the TPC and TFC in different parts of the *B. elegans* plant, including seeds, stems, leaves, and roots. TPC and TFC values are indicated as means with SDs for each solvent fraction. In this study, the concentrations of TPC and TFC in the seeds, stems, leaves, and roots of *B. elegans* were measured and presented in mg GAE/mg, as illustrated in Figure 3. Phenolic content was determined by comparing GAEs/g. Flavonoids were calculated by comparing the mg QE/100 mg. For TPC, stem extracts demonstrated the highest levels overall, especially in the methanol fraction (25.89 mg GAE/100 mg), followed by ethyl acetate (13.50 mg GAE/100 mg) and diethyl ether (12.82 mg GAE/100 mg). Root extracts ranked second in TPC, with the highest values observed in ethyl acetate (21.63 mg GAE/100 mg) and notable amounts in methanol and diethyl ether. Seed extracts showed the highest TPC in methanol (6.717 mg GAE/100 mg), whereas other solvents had lower TPC values. The leaf extracts had moderate TPC, with the highest content in methanol (7.66 mg GAE/100 mg).

**TABLE 1:** The mean of total phenolic and total flavonoid contents was mean and SD.

Sample	TPC				TFC			
	Methanol fraction ± SD	Ethyl acetate fraction ± SD	Diethyl ether fraction ± SD	Hexane fraction ± SD	Methanol fraction ± SD	Ethyl acetate fraction ± SD	Diethyl ether fraction ± SD	Hexane fraction ± SD
Seeds	0.72 ± 0.04	0.40 ± 0.01	0.23 ± 0.004	0.30 ± 0.01	1.23 ± 0.004	0.13 ± 0.005	0.10 ± 0.005	0.06 ± 0.009
Stems	1.43 ± 0.06	0.73 ± 0.10	0.69 ± 0.044	0.56 ± 0.18	0.38 ± 0.01	0.89 ± 0.14	0.60 ± 0.024	0.45 ± 0.03
Leaves	0.83 ± 0.03	0.48 ± 0.046	0.50 ± 0.017	0.25 ± 0.01	0.86 ± 0.02	0.38 ± 0.01	0.30 ± 0.015	0.25 ± 0.01
Roots	0.30 ± 0.03	0.45 ± 0.05	0.35 ± 0.007	0.23 ± 0.04	0.43 ± 0.042	0.19 ± 0.02	1.24 ± 0.115	1.22 ± 0.03

*Note:* The data are expressed as a mean (*n* = 3) ± SD.

FIGURE 3MeOHF: methanol extract fraction; EOACF: ethyl acetate extract; Et2OF: diethyl ether extract fraction; HexF: hexane fraction extract. (a) TPC was expressed in mg GAE; (b) TFC was expressed in mg QE/g.

**Figure:**
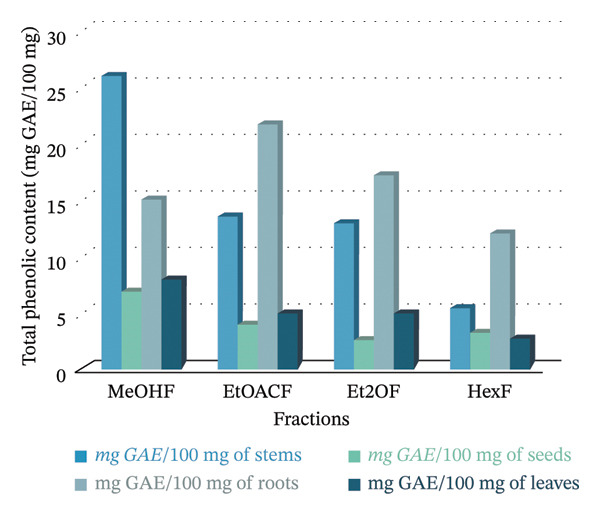
(a)

**Figure:**
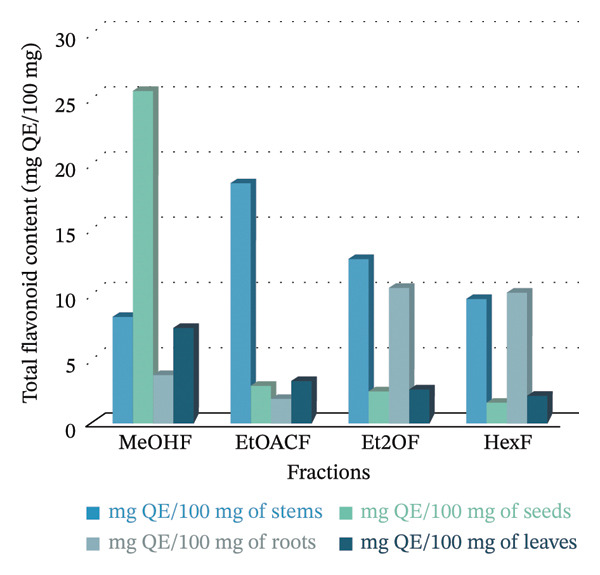
(b)

Regarding TFC, seed extracts were most prominent in the methanol fraction (25.35 mg QE/100 mg), indicating a higher flavonoid concentration than other solvents with considerably lower values. Stem extracts had the highest TFC in ethyl acetate (18.29 mg QE/100 mg), with diethyl ether (12.46 mg QE/100 mg) and hexane (9.43 mg QE/100 mg) following. Root extracts had significant TFC in diethyl ether (10.21 mg QE/100 mg) and hexane (10.04 mg QE/100 mg), whereas the methanol fraction was relatively low (3.56 mg QE/100 mg). Leaf extracts exhibited moderate TFC in methanol (7.06 mg QE/100 mg), with much lower levels in ethyl acetate (3.08 mg QE/100 mg), diethyl ether (2.46 mg QE/100 mg), and hexane (1.99 mg QE/100 mg).

### 3.3. HPLC Analysis of Phenolic Compounds

Table 2 displays the standard chromatogram values of 15 individual phenolic substances found in the seeds, leaves, stems, and roots of the *B. elegans* species. The results showed a detailed analysis of various bioactive compounds across different plant parts (seeds, leaves, stems, and roots) using three extraction methods: methanolic extraction, selective extraction, and acidic hydrolysis. In addition, the LOD and LOQ were determined to identify and quantify the phenolic compounds in the extracts (Table 3).

**TABLE 2:** Analysis of phenolic constituents of *B. elegans* by HPLC–DAD.

Phenolic compounds	Seed			Leaf			Stem			Root		
	m^1^	s^2^	a^3^	m	s	a	m	s	a	m	s	a
Gallic acid	29.37	5.72	28.46	22.03	9.19	24.83	15.64	5.00	6.77	20.27	6.87	14.04
Coumalic acid	17.42	4.50	110.35	10.03	5.93	59.48	10.83	4.47	6.25	8.307	9.41	259.23
Chlorogenic acid	11.65	7.37	5.65	300.25	46.62	13.23	116.18	8.72	6.49	23.17	24.27	16.91
Vitamin C	8.588	0.32	3.02	9.87	15.66	3.40	12.06	2.51	7.07	13.24	5.988	7.764
Caffeic acid	16.04	52.90	19.72	430.22	183.94	31.76	99.07	29.23	22.35	37.45	59.78	61.73
Syringic acid	6.46	34.42	11.30	149.84	70.12	17.27	54.19	46.60	8.69	41.69	120.83	12.01
Ellagic	1.38	ND	0.53	12.96	3.85	4.00	18.96	0.07	3.91	4.90	2.72	0.83
p‐Coumaric acid	386.70	8.772	3.93	102.77	40.08	4.06	33.19	3.78	1.194	14.34	14.15	4.38
Rutin	394.22	18.33	29.08	376.21	77.04	60.52	295.20	47.50	49.81	108.41	72.205	22.59
Ferulic acid	2612.28	337.15	2.84	913.13	504.22	46.79	891.39	67.59	10.23	387.12	100.36	35.31
Myricetin	60.14	8.11	4.87	44.53	44.71	16.72	76.41	20.50	18.79	23.73	45.86	12.37
Rosmarinic acid	1179.0	34.59	ND	419.37	141.95	1.971	171.99	62.02	3.40	71.08	77.30	10.51
Resveratrol	154.68	5.03	ND	31.88	17.41	5.48	27.04	8.72	5.08	10.81	11.92	6.85
Quercetin	9.26	ND	ND	14.59	40.53	7.15	8.915	21.36	7.019	7.11	32.18	ND
*Trans*‐cinnamic	ND	ND	ND	7.95	7.15	ND	0.83	1.118	ND	ND	0.87	ND

Abbreviation: ND = Not detected.

^1^m = methanolic.

^2^s = selective extraction.

^3^a = acidic hydrolysis.

**TABLE 3:** Analytical data for the HPLC method for the studied phenolic compounds.

No.	Compound	Retention time (min)	Detection wavelength (nm)	Equation of calibration curve	Coefficient of determination (*R* ^2^)	LOD (ug/mL)	LOQ (ug/mL)
1	Gallic acid	3.44	278	*y* = 11,041*x* − 15,175	0.9993	1.22	3.7
2	Coumalic acid	5.07	254	*y* = 10,207*x* − 26,244	0.9991	1.72	5.22
3	Chlorogenic acid	8.42	300	*y* = 4735.4*x* − 2462.7	0.9999	0.65	1.97
4	Vitamin C	9.87	254	*y* = 9515.4*x* + 4076.3	0.9998	0.87	2.63
5	Caffeic acid	10.36	300	*y* = 7777.4.4*x* − 5289	0.9991	1.95	5.93
6	Syringic acid	10.41	278	*y* = 15,364*x* − 29,142	0.9994	1.81	5.47
7	Ellagic	12.29	254	*y* = 15,719*x* + 21,189	0.9996	1.27	3.84
8	Rutin	12.48	254	*y* = 1681.1*x* − 3530.7	0.9991	2.20	6.67
9	*p*‐Coumaric acid	12.88	300	*y* = 29,210*x* + 8774.4	0.9991	1.83	5.53
10	Ferulic acid	13.67	300	*y* = 2445.4*x* − 17897	0.9993	1.97	5.97
11	Myricetin	16.31	254	*y* = 1980.5*x* − 2510.9	0.9994	1.54	4.67
12	Rosmarinic acid	16.86	300	*y* = 2901.9*x* + 2343.9	0.9995	1.35	4.09
13	Resveratrol	19.15	300	*y* = 19,296*x* − 22,779	0.9993	1.92	5.82
14	Quercetin	23.58	278	*y* = 2934.7*x* − 15,910	0.9992	1.71	5.19
15	*Trans*‐cinnamic	26.03	278	*y* = 73,869*x* + 18,870	0.9999	0.47	1.42

*Note:* Here, *n* = 3, RSD = 1.5%.

The analysis revealed that gallic acid was predominantly present in the seed extracts obtained via acidic hydrolysis (28.47 μg/g), with appreciable levels across all plant parts. Coumalic acid was most concentrated in root extracts processed by acidic hydrolysis (259.24 μg/g) and was also present in the leaf extracts (59.49 μg/g). Chlorogenic acid was the most abundant in leaf extracts obtained through methanolic extraction (300.25 μg/g), with considerable amounts also detected in stem extracts (116.19 μg/g). The highest concentration of vitamin C was observed in the leaf extracts obtained via selective extraction (15.66 μg/g). The leaf extracts processed by methanolic extraction had the highest caffeic acid content (430.22 μg/g), with notable concentrations in stems (99.08 μg/g) and roots (59.78 μg/g). Syringic acid levels were notably high in selective leaf extracts (70.12 μg/g) and root extracts (120.84 μg/g). Ellagic acid was present in lower quantities, with the highest being detected in stem extracts through methanolic extraction (18.97 μg/g). The highest concentration of *p*‐coumaric acid was found in seed extracts from the methanolic extraction (386.71 μg/g), with significant levels in leaf extracts (102.77 μg/g). Rutin was highly concentrated in both seed (394.22 μg/g) and leaf (376.22 μg/g) methanolic extracts. Ferulic acid reached a very high concentration in seed methanolic extracts (2612.28 μg/g), which was significantly present across all plant parts, especially in leaf and stem extracts. Myricetin was the most concentrated in stem methanolic extracts (76.41 μg/g), with moderate levels in other parts. Rosmarinic acid was most prevalent in seed methanolic extracts (1179.01 μg/g) and was found at various concentrations across all parts. Resveratrol was mainly detected in seed methanolic extracts (154.68 μg/g) and root extracts obtained through selective extraction (11.92 μg/g). Quercetin was the most abundant in leaf extracts obtained through selective extraction (40.53 μg/g), generally showing lower levels in other plant parts. *Trans*‐cinnamic acid was minimally present, particularly in leaf methanolic extracts (7.95 μg/g).

In this analysis, some phenolic compounds were absent from specific plant parts or extraction methods. For example, ellagic acid was undetectable in seed extracts obtained through selective extraction and acidic hydrolysis, and it was absent in the stem and root extracts produced via selective extraction. Quercetin was not found in seed extracts from selective extraction and acidic hydrolysis, nor in root extracts obtained through acidic hydrolysis. Similarly, *trans*‐cinnamic acid was absent from all seed extracts across all extraction methods. It was not detected in root extracts derived from methanolic and selective extracts or in leaf extracts subjected to acidic hydrolysis. These absences could be due to the absence of these specific compounds in the analyzed plant parts, the inability of the extraction methods to effectively isolate these compounds, or concentrations being below the detection thresholds of the analytical techniques used.

In general, methanolic extraction is more effective at yielding higher compound concentrations than selective extraction and acidic hydrolysis. Seeds were particularly rich in ferulic and rosmarinic acid, leaves were notable for high levels of chlorogenic and caffeic acids, and roots showed significant concentrations of coumalic and syringic acids under certain extraction conditions. Figure 1 shows the HPLC chromatograms of a standard mixture of analytes at 254, 287, and 300 nm.

### 3.4. GC/MS Analysis of *B. elegans* Seed Extract

**FIGURE 4:**
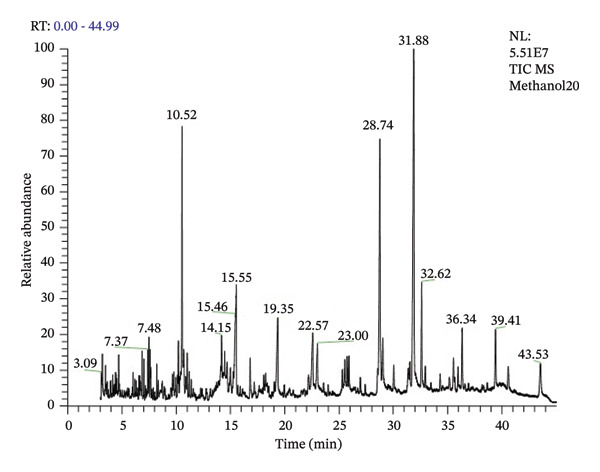
GC/MS chromatogram of *B. elegans* methanol seed extract.

**FIGURE 5:**
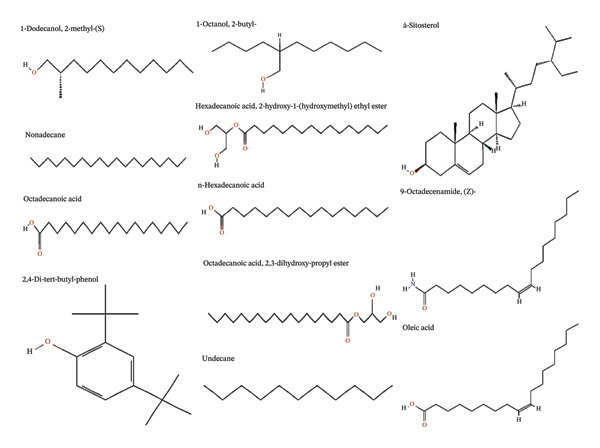
The chemical structures of compounds extracted from *B. elegans* seed.

GC–MS analysis was used to determine the phytochemical composition of the *B. elegans* seed extract. The chromatogram in Figure 4 shows the presence of seven distinct compounds. Each peak was identified by comparing the RTs and mass spectra of the reference substances with those of the software libraries. The identification and structures of the compounds were confirmed by matching the RTs and fragmentation patterns in the mass spectra with the NIST library database. Table 3 lists the bioactive compounds detected in the *B. elegans* extract, along with their chemical names, molecular formulas, and RTs from chromatographic analysis. Figure 5 illustrates the molecular structures of the bioactive compounds. These identified substances were then used in molecular docking studies (see Table 4).

**TABLE 4:** Identification of the components in *B. elegans* methanol seed extract using GC/MS.

No.	$R_{t}^{*}$	Compound name	Molecular formula	Molecular weight	SI	RSI
1	7.37	1‐Dodecanol, 2‐methyl‐(S)	C_13_H_28_O	200	818	839
2	7.48	1‐Octanol, 2‐butyl‐	C_12_H_26_O	186	827	868
3	10.52	2,4‐Di‐tert‐butyl‐phenol	C_14_H_22_O	206	902	923
4	15.55	Nonadecane	C_19_H_40_	268	801	906
5	19.35	n‐Hexadecanoic acid	C_16_H_32_O_2_	256	836	853
6	22.75	Oleic Acid	C_18_H_34_O_2_	282	847	869
7	23.00	Octadecanoic acid	C_18_H_36_O_2_	284	817	882
8	28.74	Hexadecanoic acid, 2‐hydroxy‐1‐(hydroxymethyl) ethyl ester	C_19_H_38_O_4_	330	854	867
9	31.88	Octadecanoic acid, 2,3‐dihydroxy‐propyl ester	C_21_H_42_O_4_	358	866	897
10	32.62	9‐Octadecenamide, (Z)‐	C_18_H_35_NO	281	820	883
11	39.41	á‐Sitosterol	C_29_H_50_O	414	803	826

Abbreviation: RT, retention time.

### 3.5. Molecular Docking Study

The methanol extract from the seeds contained significant compounds, including 1‐dodecanol, 2‐methyl‐(S), 1‐octanol, 2‐butyl, 2,4‐di‐tert‐butyl‐phenol, nonadecane, *n*‐hexadecanoic acid, oleic acid, octadecanoic acid, hexadecanoic acid, 2‐hydroxy‐1‐(hydroxymethyl) ethyl ester, octadecanoic acid, 2,3‐dihydroxy‐propyl ester, 9‐octadecenamide (Z), and á‐sitosterol. Table 5 shows the ΔG values of the seven ligands for the vimentin and BCL2 receptors. The binding affinity (ΔG bind) represents the strength of the interaction between a ligand and protein receptor. This interaction is influenced by the ΔG binding value, where a more negative value indicates a better fit between the bioactive compounds and various proteins, resulting in more stable binding of the ligand to the receptor.

**TABLE 5:** List of 7 known active antagonists of the vimentin and BCL2 protein, along with their binding affinities determined through molecular docking analysis.

Compound	PubChem ID	Binding affinity toward vimentin	Binding affinity toward BCL*2*
A‐Sitosterol	71752209	−7.2	−8.3
Tetradecanoic acid	11,005	−4.3	−4.7
Undecane	14,257	−3.8	−4
Oleic acid	445,639	−5.1	−5.3
9‐Octadecenamide, (Z)	5283387	−3.5	−5.8
2,4‐Di‐tert‐butylphenol	7311	−6.7	−6.4
n‐Hexadecanoic acid	985	−5.1	−4.8

The re‐docking data confirmed the accuracy of the docking methods and parameters, as the ligands were found to bind closely to their actual conformations on the targets. Figure 6 illustrates the 3D interaction between the ligand á‐sitosterol and the vimentin receptor, showing both the overall interaction and the specific atoms involved, along with the bonds formed between the ligand and the receptor protein. Similarly, Figure 7 depicts the 3D interaction between the ligand á‐sitosterol and the BCL2 receptor, including the interacting atoms and the ligand–receptor protein bond. It is clear from Table 5 that the á‐sitosterol compound has the lowest binding affinity to vimentin and BCL2 receptors, indicating its potential anticancer activities. I think in dissection.

FIGURE 6(a) The 3D interaction between the ligand A‐sitosterol and the receptor vimentin; (b) the interacting atoms and the bond formed between the ligand and the receptor protein in receptor.

**Figure:**
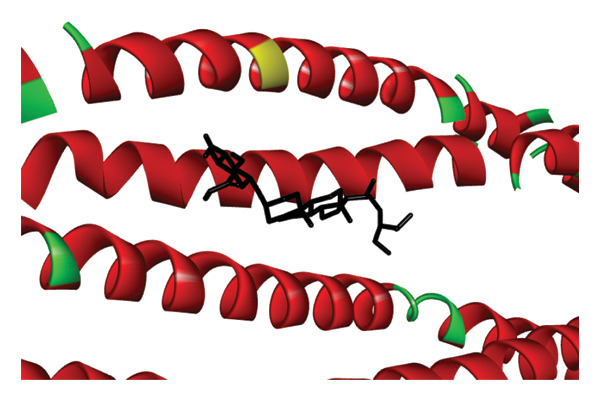
(a)

**Figure:**
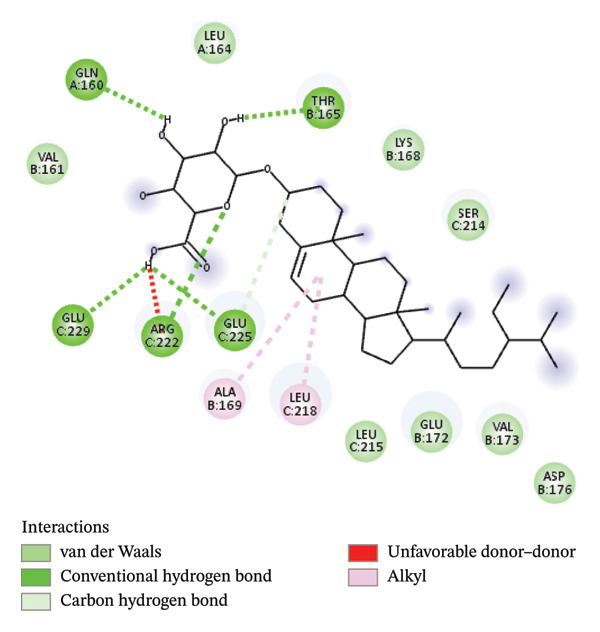
(b)

FIGURE 7(a) The 3D interaction between the ligand A‐sitosterol and the receptor BCL2; (b) the interacting atoms and the bond formed between the ligand and the receptor protein in the receptor.

**Figure:**
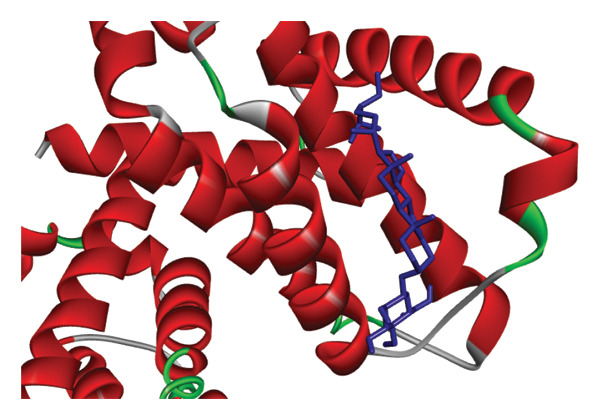
(a)

**Figure:**
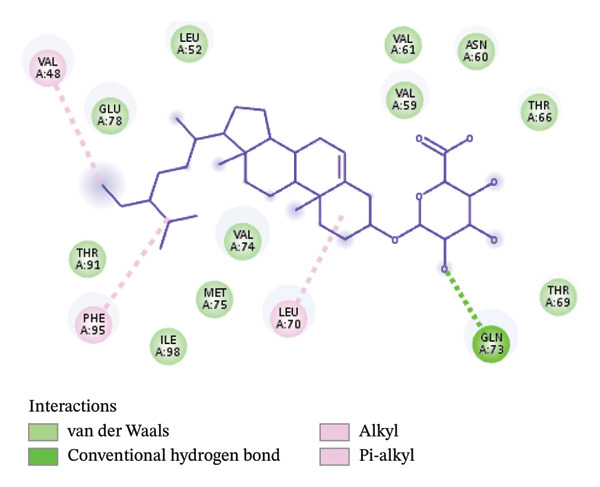
(b)

### 3.6. Impact of *B. elegans* Methanolic Extract on the Proliferation of Various Cancer Cell Lines

The cytotoxic effects of various *B. elegans* extracts were evaluated using MCF‐7, MDA231, and HepG2 cell lines. The cells were treated with serial dilutions of *B. elegans* extracts prepared in methanol, ethyl acetate, diethyl ether, and hexane. The percentage viability of HepG2, MDA‐MB‐231, and MCF‐7 cells after 24 h of treatment with different *B. elegans* extracts is shown in Figure 8. The results indicated that *B. elegans* extracts from the seeds, leaves, and roots exhibited dose‐dependent cytotoxicity against MCF‐7, MDA‐MB‐231, and HepG2 cell lines, with cell viability decreasing as the extract concentration increased. The study showed that HepG2 cells, a type of human liver cancer, were significantly inhibited by the methanol extracts of leaves and roots, with inhibition rates of 82.5% and 78%, respectively, at a concentration of 1000 μg/mL. The diethyl ether fraction of the stem extract demonstrated strong cytotoxicity, inhibiting 82.06% of the cells at 1000 μg/mL. Additionally, the seed extract also exhibited cytotoxic effects, inhibiting 77.2% of HepG2 cell growth at the same concentration. For the MCF‐7 cell line, the ethyl acetate extract from the roots showed the most potent cytotoxic activity, reducing cell viability by 83.55%, whereas the seed extract reduced viability by 67.5% at a 1000 μg/mL concentration. At the same concentration, the diethyl ether extract of the leaves and stems inhibited cell viability by 81.72% and 66.61%, respectively. For the MDA‐MB‐231 cell line, the hexane extracted from leaves, seeds, and stems at a concentration of 1000 μg/mL showed the strongest cytotoxic effects, reducing cell viability by 88.8%, 77.6%, and 75.2%, respectively. Similarly, the methanol extract from roots, at the same concentration, also displayed considerable cytotoxicity, decreasing cell viability by 77.3%. When present at a 1000 μg/mL concentration, all extracts exhibited cell growth inhibition ranging from 62% to 88.8%. At low concentrations of 62 and 125 μg/mL, cytotoxicity did not affect the viability of the extracts. The hexane extract had a less pronounced effect on the viability of the HepG2 and MCF‐7 cells. The inhibitory half‐maximum concentration (IC50) was also calculated from the curves.

FIGURE 8The percentage of cell viability at various concentrations μg/mL of *B. elegans* in different solvent extracts as: (a) MCF‐7 cell line; (b) HePG2 cell line; (c) MDA‐MB‐231 cell line.

**Figure:**
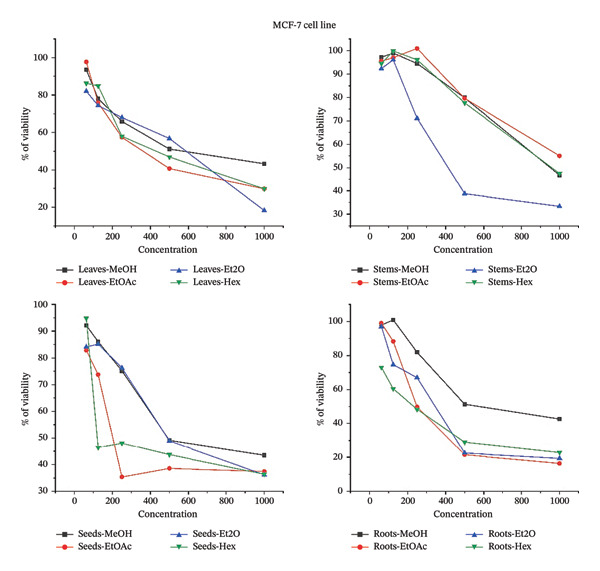
(a)

**Figure:**
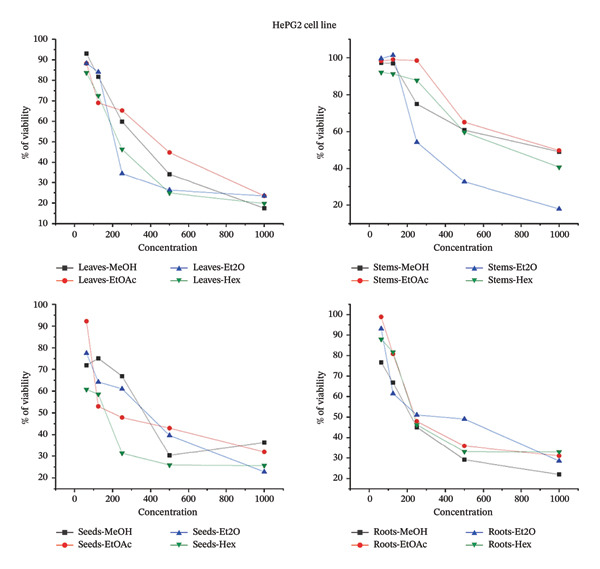
(b)

**Figure:**
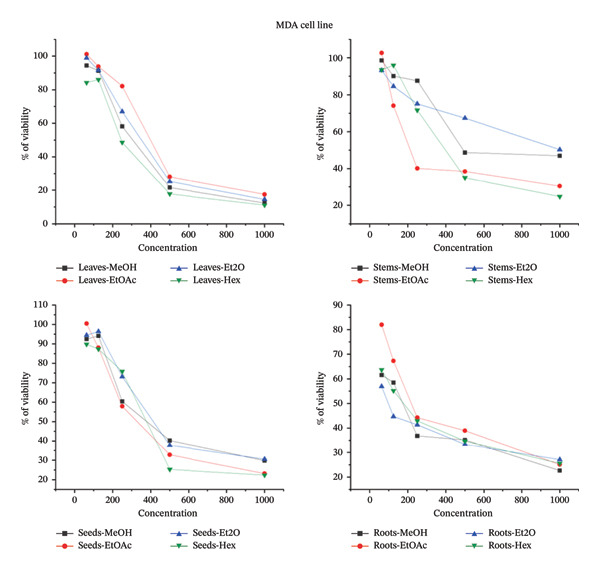
(c)

The cell death IC_50_ was also calculated from the curves. The IC_50_ potential cytotoxicity against *B. elegans* for each cell line, HepG2, MCF‐7, and MDA231 is indicated in Table 6.

**TABLE 6:** The extract IC_50_ potential cytotoxicity against *B. elegans* for each cell line. MeOH: methanol, EtOAc: ethyl acetate, EtO_2_: diethyl ether, Hex: hexane.

Extract	IC_50_		
	HepG2	MCF‐7	MDA231
Seed‐MeOH	319	275	459.2
Seed‐EtOAc	305	248	369.7
Seed‐EtO_2_	334	559	462.4
Seed‐Hex	139	218	433.3

Stem‐MeOH	935	1538	929.5
Stem‐EtOAc	1114	1602	320.7
Stem‐EtO_2_	393	525	986.5
Stem‐Hex	871	1244	456

Leaf‐MeOH	378.3	607.5	315.5
Leaf‐EtOAc	420.5	403	401.9
Leaf‐EtO_2_	262.8	455.2	360.4
Leaf‐Hex	250.4	398.5	285.1

Root‐MeOH	214.7	668.8	178.8
Root‐EtOAc	323.2	315.5	222.7
Root‐EtO_2_	388.5	300.6	145.7
Root‐Hex	350.2	191.1	160.1

## 4. Discussion

Various traditional medicines are currently available. Still, only a limited number of chemical and pharmacological studies have been conducted on various species of the *Boerhavia* genus, such as *Boerhavia plumbaginea, Boerhavia chinensis, Boerhavia repens, Boerhavia diffusa, Boerhavia erecta*, and *Boerhavia elegans*. Therefore, modern pharmacochemical research is needed to investigate the bioactivity associated with traditional knowledge. Boerhavia has gained increasing attention from researchers due to its diverse pharmacological and biological activities, suggesting its therapeutic potential. For instance, according to a study, *B. diffusa* methanolic extract leaves exhibited antibacterial properties and significant activity against *Staphylococcus aureus*, a harmful bacterium (Satish and Girish) [20, 41]. In 2010, Ramazani and colleagues in Iran carried out a study that showed the antimalarial properties of *B. elegans*, marking one of the first studies to demonstrate the effectiveness of the plant against malaria [42]. Traditional medicine practices have also indicated that *B. elegans* may benefit from various other health issues, including painful menstruation, urinary tract problems, inflammation, jaundice, intestinal infections, and overall weakness [40]. In a study conducted by Sadeghi et al. in Iran, the antioxidant properties of fruit, stem, and leaf extracts were assessed using chloroform, ethyl acetate, methanol, and water. The results indicated that the methanol extract had the highest DPPH value compared to the extracts obtained using the other solvents [19, 43]. This agrees with the findings of this study, as the methanol extract of *B. elegans* had a higher percentage of DPPH scavenging activity than the ethyl acetate extract and hexane. The results demonstrated that methanol exhibits a high rate of scavenging activity, which can be ascribed to the positive association between the quantity of total phenols and antioxidant activity. Zhang et al. demonstrated that *Boerhavia elegans* effectively neutralizes free radicals by converting the stable DPPH radical into a yellow diphenyl picryl hydrazine compound [43, 44].

Medicinal herbs and edible plants contain phenolic chemicals and important secondary metabolites. An aromatic ring joined to a hydroxyl group is the defining feature of these compounds. There is an extensive list of phenolic compounds, including cinnamic acid, coumarins, benzoic acid, tannins, lignins, and flavonoids [45]. Phenolic compounds and flavonoids are recognized for their diverse biological activities, including antioxidant and anti‐inflammatory properties [46]. Recent research indicates that diets rich in polyphenolic compounds may play a vital role in managing oxidative stress‐related disorders because of their antioxidant properties [47]. Consequently, the polyphenolic constituents present in the entire *B. elegans* plant have the potential to alleviate conditions associated with oxidative stress. The results from the total phenols and flavonoids assays indicated that the amounts present in the extracts of *B. elegans* varied, suggesting that different solvent systems vary significantly in their efficiency in extracting phenolic and flavonoid compounds from various parts of *B. elegans*. This variability can be attributed to two factors: the use of various solvents in the extraction procedure and the antioxidant activity of the extracts. The choice of solvent can influence the efficiency of phenol extraction from the plant material, leading to variations in the phenolic content of the extracts [48]. The methanol extract had the highest phenol value, which was consistent with its antioxidant results, whereas the hexane extract had the lowest phenol value. This difference is due to the low polarity of the solvent, which amounted to 26.55 mg [49, 50]. Methanol consistently extracts high amounts of phenolics and flavonoids, especially in seed and stem extracts, making it the most effective solvent for these compounds. The high extraction efficiency of methanol is primarily attributed to its polar nature, strong hydrogen‐bonding ability, and miscibility with water, which enable it to dissolve a wide range of polar phytochemicals, particularly phenolic acids and flavonoids—the main contributors to antioxidant activity. Its small molecular size also facilitates better penetration of plant tissues, improving mass transfer and overall extraction yield.

Although ethyl acetate and diethyl ether also showed a considerable ability to extract phenolic and flavonoid compounds, particularly from the stem and root tissues, their performance was lower than that of methanol due to their intermediate polarity. In contrast, hexane, a nonpolar solvent, was the least effective in extracting these compounds, reflecting its limited capacity to dissolve polar antioxidants [51–55].

Among the phenolic compounds identified by HPLC–DAD, ferulic acid was the most abundant constituent, followed by rosmarinic, *p*‐coumaric, rutin, and caffeic acids. The high abundance of ferulic acid is particularly significant, as it exhibits strong antioxidant and free radical scavenging properties, neutralizes reactive oxygen species, inhibits lipid peroxidation, and enhances the activity of endogenous antioxidant enzymes such as superoxide dismutase and catalase. Beyond its antioxidant role, ferulic acid demonstrates anti‐inflammatory and anticancer potential by modulating key signaling pathways, including NF‐κB and PI3K/AKT, leading to the suppression of oxidative stress and induction of apoptosis in cancer cells, as well as contributing to cardiovascular and metabolic protection [56]. Similarly, rosmarinic acid, a potent polyphenolic compound, enhances antioxidant defense through activation of the Nrf2 pathway and chelation of metal ions, while its anticancer activity involves modulation of NF‐κB and PI3K/AKT pathways, inhibition of tumor proliferation, and protection against oxidative DNA damage [57]. *p*‐Coumaric acid also exhibits strong antioxidant, anti‐inflammatory, and anticancer activities by scavenging free radicals, inhibiting lipid peroxidation, and suppressing NF‐κB and COX‐2 pathways, while inducing apoptosis and regulating PI3K/AKT and MAPK signaling [58]. In addition, rutin, a flavonoid glycoside of quercetin, contributes to antioxidant and anti‐inflammatory activity by enhancing enzymatic antioxidant defenses and modulating inflammatory mediators such as TNF‐α and IL‐6, although its bioavailability can be improved through conversion to isoquercitrin [59]. Caffeic acid further reinforces these effects through its dual hydroxyl groups, which enable effective free radical scavenging, inhibition of lipid peroxidation, and suppression of COX‐2 and NF‐κB expression, resulting in reduced oxidative stress and inhibition of cancer cell proliferation [60]. Collectively, the predominance of these bioactive phenolics explains the strong antioxidant and cytotoxic activities observed in *Boerhavia elegans* extracts and highlights the crucial role of solvent polarity—particularly methanol—in maximizing their extraction efficiency.

In a study by Sadeghi et al. [19], the aerial parts of *Boerhavia elegans* at a concentration of 50 μg/mL showed no inhibitory effect on PC3 cells, whereas doxorubicin completely inhibited cell growth at 10 μg/mL. Similarly, *B. elegans* (50 μg/mL) exhibited only 15.14% inhibition of the MCF‐7 cell line, compared to 95% inhibition by doxorubicin (10 μg/mL) [19]. These findings differ from the results of the present study, which demonstrated approximately 83.5% inhibition at 1000 μg/mL, indicating a stronger cytotoxic potential under the tested conditions.

The molecular docking results provide additional support for the experimental findings and help clarify the possible mechanisms underlying the biological activities of *Boerhavia elegans* extracts. Compounds identified by GC–MS—particularly phytol, stigmasterol, hexadecanoic acid, and β‐sitosterol—showed strong binding affinities toward BCL‐2 and vimentin, proteins that regulate apoptosis, cell proliferation, and metastasis. These interactions are consistent with the cytotoxic activity observed experimentally, suggesting that these molecules may induce apoptosis and inhibit cancer cell growth by interfering with these targets. The docking data also complement the antioxidant results, as several of the identified compounds contain hydroxyl and unsaturated groups that can donate hydrogen atoms and stabilize free radicals. Together, these findings demonstrate that the phytochemicals identified in *B. elegans* contribute to its antioxidant and cytotoxic potential and provide molecular‐level evidence supporting its therapeutic relevance.

β‐Sitosterol, a major phytosterol structurally similar to cholesterol, demonstrates significant pharmacological potential, particularly in cancer therapy. Nandi et al. highlighted its anticancer properties across several cancer types, including leukemia, lung, and breast cancers. β‐Sitosterol modulates key cancer‐related pathways involved in cell proliferation and apoptosis and may enhance the efficacy of conventional therapies. Although less potent than standard chemotherapeutic agents, its low toxicity and favorable pharmacological profile suggest it as a promising and safe candidate for cancer treatment. In addition to β‐sitosterol, other major constituents detected by GC–MS exhibited relevant molecular interactions with cancer‐related proteins [61]. Phytol, a diterpene alcohol, formed stable hydrogen bonds and hydrophobic interactions with the BCL‐2 active site, suggesting a role in apoptosis regulation. Stigmasterol displayed docking affinities comparable to β‐sitosterol, supporting its potential involvement in cell membrane stabilization and antiproliferative activity. Hexadecanoic acid and 9,12‐octadecadienoic acid established hydrophobic contacts with key amino acid residues in vimentin, indicating potential interference with cytoskeletal organization. These results suggest that the cytotoxic activity of *B. elegans* extracts may arise from the synergistic effects of multiple bioactive constituents rather than the action of a single compound.

## 5. Conclusions

Our study highlights the significant medicinal potential of *Boerhavia elegans*, a plant long valued in traditional medicine. We evaluated the cytotoxicity, antioxidant properties, and contents of phenolic compounds and flavonoids in various plant parts (seeds, leaves, stems, and roots). The findings revealed that the polar fractions exhibited strong antioxidant activity, with the leaves showing the highest efficacy. Methanol proved to be the most effective solvent for extracting phenolic compounds and flavonoids from most parts of the plant, while ethyl acetate and diethyl ether were effective for specific parts. We successfully identified and quantified the phenolic compounds and flavonoids using UHPLC. Notably, *B. elegans* extracts showed promising cytotoxic effects on liver and breast cancer cell lines, with the lowest IC_50_ values observed in the hexane and diethyl ether extracts of the seeds and roots. These findings underscore the potential pharmacological applications of *B. elegans*, particularly in cancer treatment.

The overall findings reveal a strong relationship between the phytochemical composition and the biological activities of *Boerhavia elegans* extracts. The high antioxidant potential observed, particularly in the methanolic and ethyl acetate extracts, can be attributed to their richness in phenolic and flavonoid compounds, such as caffeic acid, syringic acid, and ferulic acid. In contrast, the GC–MS‐identified constituents—including phytol, hexadecanoic acid, and stigmasterol—demonstrated notable molecular docking affinities toward cancer‐related protein targets, suggesting their potential role in the observed cytotoxic activity. These results collectively indicate that *B. elegans* is a promising source of natural antioxidants and cytotoxic agents, supporting its traditional medicinal use and highlighting its potential for future therapeutic applications.

## Author Contributions

Tahreer M. Al‐Raddadi: investigation, writing–original draft, formal analysis, and investigation; Saleh O. Bahaffi: project supervision and conceptualization; Lateefa A. Alkhateeb: project co‐supervisor and methodology; Abdulaziz A. Kalantan: formal analysis; Ahmed M. Adam: software; Torki Zughaibi: visualization; and Ehab M. M. Ali: visualization and review and editing.

## Funding

No funding was received for this research. Open Access publishing was facilitated by the Deanship of Scientific Research (DSR) at King Abdulaziz University, as part of the Wiley – King Abdulaziz University agreement.

## Disclosure

All the authors have read and agreed to the published version of the manuscript.

## Ethics Statement

The authors have nothing to report.

## Consent

The authors have nothing to report.

## Conflicts of Interest

The authors declare no conflicts of interest.

## Data Availability

The data presented in this study are available upon request from the authors.
